# Geomagnetic disturbances may be environmental risk factor for multiple sclerosis: an ecological study of 111 locations in 24 countries

**DOI:** 10.1186/1471-2377-12-100

**Published:** 2012-09-24

**Authors:** Seyed Aidin Sajedi, Fahimeh Abdollahi

**Affiliations:** 1 Neurology Department, Golestan Hospital, Ahvaz Jundishapur University of Medical Sciences, Ahvaz, Iran; 2 Internal Medicine Department, Golestan Hospital, Ahvaz Jundishapur University of Medical Sciences, Ahvaz, Iran

**Keywords:** Geomagnetic disturbance, Hypothesis, Latitudinal gradient, Multiple sclerosis, Prevalence

## Abstract

**Background:**

We noticed that a hypothesis based on the effect of geomagnetic disturbances (GMD) has the ability to explain special features of multiple sclerosis (MS). Areas around geomagnetic 60 degree latitude (GM60L) experience the greatest amount of GMD. The easiest way to evaluate our hypothesis was to test the association of MS prevalence (MSP) with angular distance to geomagnetic 60 degree latitude (AMAG60) and compare it with the known association of MS with geographical latitude (GL). We did the same with angular distance to geographic 60 degree latitude (AGRAPH60) as a control.

**Methods:**

English written papers with MSP keywords, done in Europe (EUR), North America (NA) or Australasia (AUS) were retrieved from the PubMed. Geomagnetic coordinates were determined for each location and AMAG60 was calculated as absolute value of numerical difference between its geomagnetic latitude from GM60L. By an ecological study with using meta-regression analyses, the relationship of MSP with GL, AMAG60 and AGRAPH60 were evaluated separately. MSP data were weighted by square root of number of prevalent cases. Models were compared by their adjusted R square (AR^2^) and standard error of estimate (SEE).

**Results:**

111 MSP data were entered in the study. In each continent, AMAG60 had the best correlation with MSP, the largest AR^2^ (0.47, 0.42 and 0.84 for EUR, NA and AUS, respectively) and the least SEE. Merging both hemispheres data, AMAG60 explained 56% of MSP variations with the least SEE (R = 0.75, AR^2^ = 0.56, SEE = 57), while GL explained 17% (R = 0.41, AR^2^ = 0.17, SEE = 78.5) and AGRAPH60 explained 12% of that variations with the highest SEE (R = 0.35, AR^2^ = 0.12, SEE = 80.5).

**Conclusions:**

Our results confirmed that AMAG60 is the best describer of MSP variations and has the strongest association with MSP distribution. They clarified that the well-known latitudinal gradient of MSP may be actually a gradient related to GM60L. Moreover, the location of GM60L can elucidate why MSP has parabolic and linear gradient in the north and south hemisphere, respectively. This preliminary evaluation supported that GMD can be the mysterious environmental risk factor for MS. We believe that this hypothesis deserves to be considered for further validation studies.

## Background

In spite of many efforts since the time of rendering Multiple Sclerosis (MS) as a disease, its etiology remains unclear. Although it is accepted that auto-reactive lymphocytes play essential role in MS, the cause of this auto-reactivity and its relapsing-remitting course are unknown [1]. Up to now, genetic studies could not determine specific genes as the cause of MS and studies on monozygotic twins demonstrated that the role of genes in the susceptibility to MS does not exceed 30% [2].

Researchers always indicate that the major role in MS pathogenesis is attributed to the effect of environmental factor(s) on the basis of a genetical susceptibility [1]. Specific epidemiological features of MS, such as significant latitudinal gradient of prevalence, the effect of migration, and the effect of month of birth on alteration of the risk of MS, all indicate to the presence of an environmental factor that should be strongly related to time, location and geophysical features. It is repeatedly tested and accepted that MS prevalence has a latitudinal gradient and its prevalence has a positive relation to the distance from the equator [3]. Based on this fact, all efforts have been toward finding a hypothesis that explains MS etiology by an environmental factor that change in agreement with this feature. According to this opinion, the recent hypothesis that tries to explain MS prevalence distribution and etiology by the possible immunomodulatory effect of vitamin D [4] seems to be reasonable, because of the common belief that production of vitamin D is related to the amount of received solar ultra-violet radiation and expected to have a latitudinal gradient.

But there is an important feature in MS prevalence that seems to be neglected by researchers who seek the key of MS explanation by vitamin D hypothesis (VDH). This fact is that MS prevalence gradient is different in north and south hemispheres and is not linear everywhere. Based on epidemiological data, this gradient is parabolic in north hemisphere, while seems linear in the south hemisphere. This feature was identified from the first reports of this gradient [5] up to the most recent study [3].

Regarding to this issue and other epidemiological features of MS, we tried to investigate if there is another environmental factor that give a potentially better explanation about MS distribution. To achieve this aim, we reviewed possible environmental factors from space to ground level and checked whether their effects on MS epidemiology were studied or not. Interestingly, many factors including extra-terrestrial phenomena such as cosmic ray to solar irradiation, and terrestrial factors such as temperature, humidity, sanitation and steel, food or energy consumption have been investigated [4,6-9]. Considering this list, we found that the possible effect of geomagnetic disturbances (GMD), a phenomenon related to interactions of space-weather situations and magnetosphere, on this disease was not concerned up to now. It is not surprising because the concept of space-weather is relatively new and the main researches about this phenomenon have been done in recent four decades [10]. Moreover, as geomagnetic field (GMF) and its disturbances are categorized as very low magnetic field (VLMF) without thermal and ionizing effect, their effects on physiological and pathophysiological issues, in comparison to other environmental factors, have generally been neglected by most biologists [11].

After comparing the various features of GMD and evaluating various evidences of effects of this phenomenon on living beings, we found that a hypothesis based on GMD has the ability to give reasonable answer to several questions about MS.

Because neuroscientists and neuroepidemiologists are among the main target population of this hypothesis and they may be unfamiliar with details of space-weather and physics of GMD, a brief review about their features has put the first.

### Basics of Space-weather and GMD

Space around our planet is not empty and the Earth is immersed in the solar energetic charged particles. Space-weather defined as the conditions in space that affect Earth, consequences of flowing ionized particle of the solar wind against GMF [10]. However GMF acts like a shield and deflect most of the solar charged particles, it is also impressed and altered by solar wind [10]. These GMF alterations are called GMD. GMF has different strength and direction in different areas of the Earth. Its magnitudes vary from ~35000 nano Tesla (nT) in equator to ~70000 nT in the magnetic poles [11]. Several *periodic variations* have also been shown in GMF that most of them are related to various solar cycles, especially 11 year cycle, and others are related to Earth’s rotation and situation in its orbit [11].

**Figure 1:**
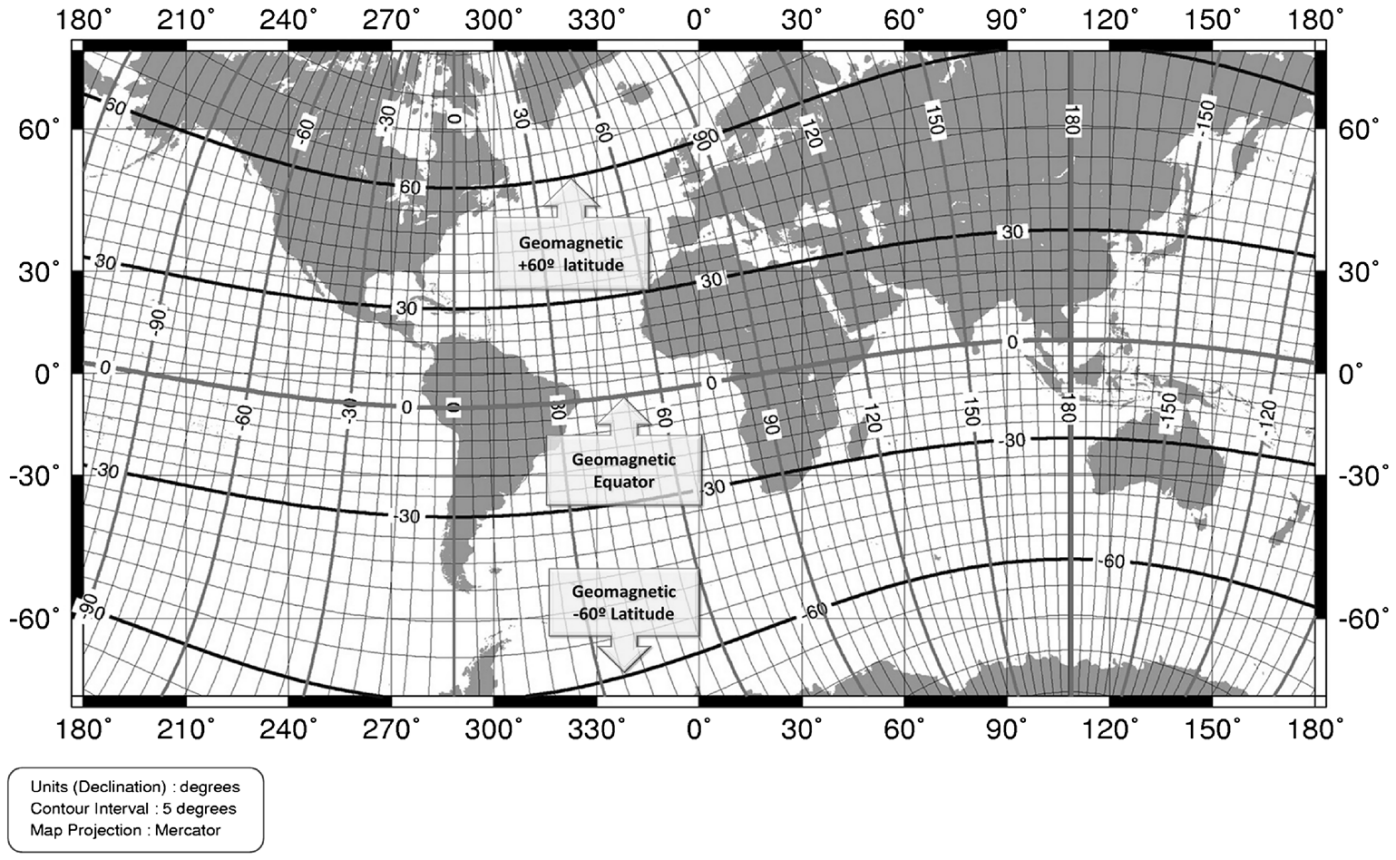
**Geomagnetic coordinates in comparison to geographic coordinates.** Note: curve lines indicate geomagnetic latitudes and longitudes. Straight lines illustrate geographic latitudes and longitudes. Reproduced by the kind permission of National Geophysical Data Center [13].

For quantifying GMD, several indices such as Planetary K index (Kp) and Planetary A index (Ap) were defined. Kp is a quasi-logarithmic scale for summarizing global geomagnetic activity in the range 0–9, which 1 and 2 being quiet state, 3 and 4 indicating unsettled and 5 or more illustrate geomagnetic storm [12]. Like geographical coordinates that are defined among Earth’s geographical poles, there are geomagnetic coordinates that are defined on the basis of Earth’s magnetic dipole. Regarding the fact that the location of magnetic dipoles are different from geographical ones and the fact that magnetic dipoles are not located completely antipodal and their locations change slowly, geomagnetic latitude and longitude of every location are completely different from its geographical coordinates (Figure 1). It is a very important note because the rate of magnetic field changes in every area on the ground level is associated with its location in the geomagnetic coordinate.

Solar activities and inter-planetary magnetic field provoke GMD by causing two different but related phenomena, including magnetospheric substorms and geomagnetic storms. Magnetospheric substorms occur frequently and disturb high latitudes of both hemispheres within ring shaped area known as auroral oval*,* and can cause up to 2000 nT GMD for few hours [14]. Auroral oval lies at geomagnetic latitude ~63° to ~75° of both hemispheres in quiet geomagnetic situations. Equatorward boundary of auroral ovals expand during unsettled situations, i.e. Kp = 3 and 4, to geomagnetic ~60° and ~58° latitude, respectively [15].

In the other hand, geomagnetic storms are less frequent but global phenomena and have impact on low and mid-latitudes, as well as high latitudes. They cause up to 400 nT GMD and last for few days [14]. During their occurrence and relative to the degree of GMD, Kp index varies among 5 to 9. It should be noted that during a geomagnetic storm, substorms also occur and the location of the equatorward boundary of auroral ovals depend on the severity of disturbances. For example, they may expand to geomagnetic ~48° latitude in Kp = 9 situation [15].

### The Hypothesis

A good hypothesis about MS should be able to give an acceptable explanation about its pathophysiology, behavior and special distribution.

In recent decades, because of vast development of wireless communication devices, concerns about the effect of VLMF on health have raised and many studies had been conducted toward them. Nevertheless, the magnitude and frequency of wireless appliance electro-magnetic fields are different from GMF and therefore, finding studies about the effect of magnetic field with same characteristics as GMF was very difficult. However such studies were sparse, in this section we tried to describe that GMD hypothesis has the potential ability to explain some aspects of MS pathophysiology and behavior.

Even with its low magnitude, GMF affects living beings. It was shown that in a weakened GMF situation, by using shielded chamber, hormonal disturbances occur in animal models, especially in the blood level of epinephrine, histamine and serotonin. Plants also are influenced by it and weakened GMF can cause changes in root meristems and subcellular structures like mitochondria [11]. We know that some species sense GMF, probably by presence of magnetites, i.e. ferromagnetic particles in their central nervous system (CNS), and use it for orientation and migration [11]. It was shown that human brain also contains magnetites [16] and it was proposed that observed increases in stress hormones, heart rate, and the amount of myocardial infarctions during geomagnetic storms may induced by causing an adaptive stress reaction through the effect of GMD on brain magnetosomes [17,18]. Accordingly, histochemical finding about the presence of considerable iron deposits within myelin loops [19] and evidences from imaging technics about increased iron deposits in subcortical gray matters of MS patients [20], in addition to some results about greater incidence of cardiovascular diseases among MS patients [21], all may be regarded as indirect clues of a probable relation among the effects of GMD on brain magnetosomes and pathogenesis of MS. However, it should be reminded that we don’t know how amount of these iron deposits are in the form of magnetite.

Regardless of these facts, any hypothesis about MS should have the ability to describe why and how adaptive immune system is activated in a relapsing-remitting manner against myelin that is an immune privileged component [22,23]. CNS is regarded as the most sensitive organ to GMF and some studies demonstrated that CNS reactions to magnetic field resulted mainly from magnetic field effects on glial cells and especially blood brain barrier [11] that are the main participants in MS pathophysiology.

However migration of myelin-specific T cells from systemic circulation to CNS has a great role in MS, their sole presence is not more regarded as the main cause of MS; because their presence was also demonstrated in healthy individuals. Rather, what is considered to be important is the activation state of these cells [22]. A naïve T cell, primarily for transforming to effector or memory T cell, needs two activator signals: peptide/major histocompatibility complex (MHC) and co-stimulatory signal. Then this effector cell for activation needs only the signal one [22]. These signals affect membrane signal transduction systems and by starting a cascade of reactions and production of messenger molecules, finally change gene expression and cell differentiation. After all, adhesion and entering to the CNS are necessary for activated T cells to be capable of exertion an inflammatory response [22,24].

There are evidences that adaptive immune system can be affected by VLMF. Magnetic field as low as GMF can significantly change lymphocyte Ca^2+^ uptake [25]. In addition, through three proposed mechanisms, GMF can change leukocyte behavior, activation and adhesion by inducing the membrane-mediated signal transduction cascades, like the time that a ligand-receptor interaction activates the cell [24-27]. Those mechanisms include changes of ion flux, especially Ca^2+^ across cell membrane, cyclotron resonance and dissociation of protein-ion complex by changing quantum states of ions in their structures in the membrane proteins [24]. There are also evidences that magnetic fields can enhance release of reactive oxygen species by T cells and macrophages [26]. Regarding these facts, we can see that GMD has the potential features to provide the essential neuroimmunological context of MS. Therefore, as the core of our hypothesis we assumed that vulnerable individuals, based on their genetical susceptibility of cell response to magnetic fields, would suffer from MS attacks in the geographical locations and time periods that GMD matches with the sensitivity of their adaptive cell immunity and lasts enough to stimulate various elements of this system for entering to CNS and activating without the presence of a co-stimulatory signal. This viewpoint can explain why the disease often has a relapsing remitting nature.

### Testing by an ecological study

**Figure 2:**
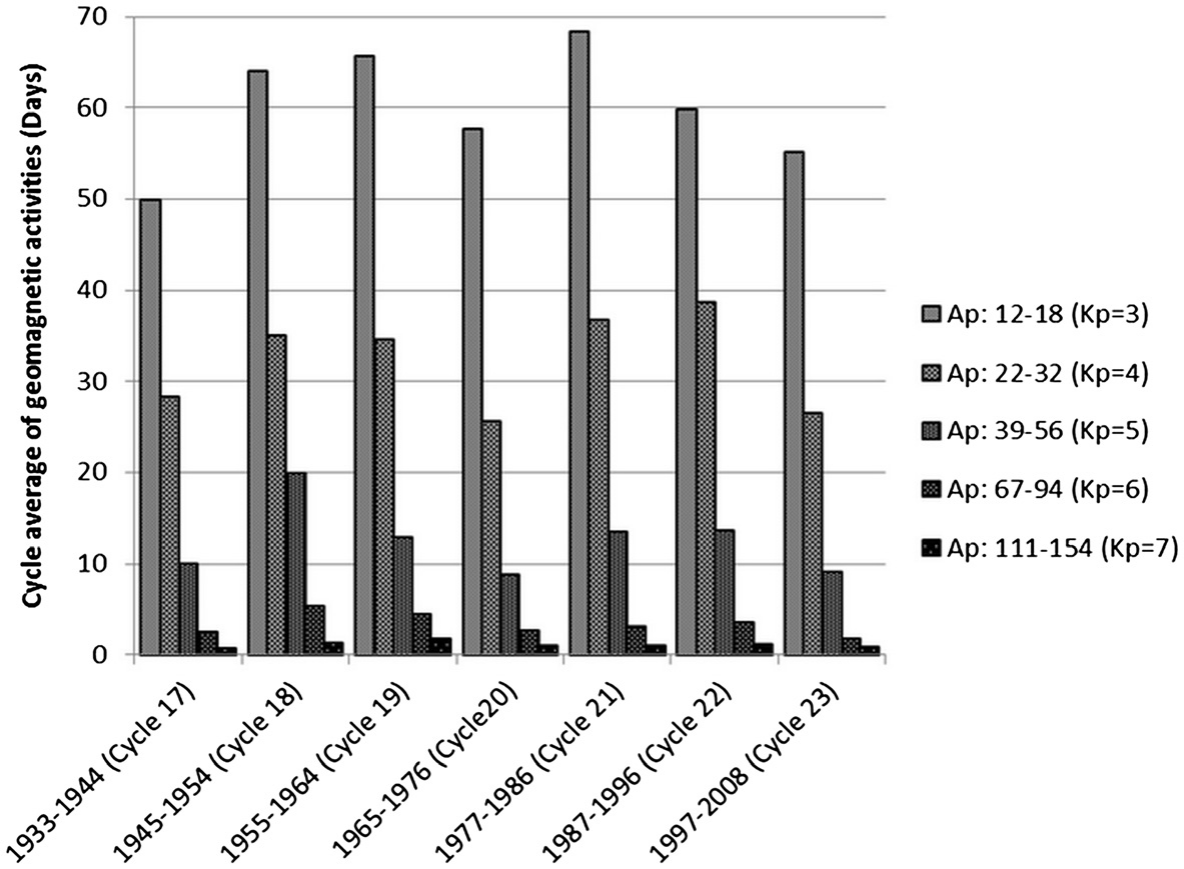
**Cycle average of number of days that has been spent in each space-weather situation during solar cycle 17 to 23.** Note: Cycle average was calculated based on daily Ap index, but because the location of auroral oval can be determined by Kp, measurements were interpreted as equal amounts of Kp. Constructed based on the Kp and Ap data from National Oceanic and Atmospheric Association (NOAA) [12] by the kind permission of NOAA.

After structuring GMD hypothesis, we planned to test it. We explained in previous sections that auroral oval boundaries are areas that more frequently and with the greatest magnitude are affected by GMD. As is obvious in Figure 2, most of disturbed days are elapsed in Kp = 3 situation in each solar cycle. In such a situation, the equatorward border of auroral oval locates upon geomagnetic ~60 latitude in both northern and southern hemispheres [15]. This means that all living beings in these areas experience more geomagnetic disturbances in comparison to other parts.

Regarding to these facts, the easiest but indirect way to evaluate the possible association of MS with GMD seemed to check the correlation of disease prevalence with *angular distance to geomagnetic 60° latitude *(AMAG60) as the border of the most affected area by space-weather through an ecological study and to compare it with the well-known association of MS with geographical latitudes, i.e. the angular distance from geographical equator. Therefore, we designed an ecological study with using meta-regression analysis on the prevalence studies of MS to check such a correlation. To have controls, we considered to check likewise correlations with angular distance to geographical 60 latitude (AGRAPH60) and geomagnetic latitudes.

## Methods

### Search strategy and selection criteria

As the most accurate MS prevalence studies have been done in western countries, we retrieved English written papers with the keywords of “prevalence” or “epidemiology” and “multiple sclerosis” in the title, published since 1980 up to the 2010, from the PubMed that were done in western countries (Additional file 1:Appendix 1. Search strategy ). In addition, authors combined articles from their archive to results retrieved from the PubMed. Our definition of western countries includes countries of *western European and other states regional group* of United Nation [28]. MS prevalence data including the location of study, the year of estimation of prevalence, the number of prevalent cases and calculated MS prevalence per 100000 were extracted from included studies. Only MS prevalence data were entered in the analyses that their original study used an approved MS diagnostic criterion with more than 20 prevalent cases and clearly indicated the number of prevalent cases. For studies that used patients’ self-report of having MS, according to their neurologists’ diagnosis, we accepted the data only if the study population were such large, i.e. at least one million or more, that choosing self-report method seemed practically inevitable and reasonable.

In the cases that several studies reported prevalence in a same location, only the latest report was entered.

For each entry, the geographical latitude and longitude were retrieved from coordinate index list of a geographical textbook [29]. In the cases that the study area involves population of a political region, i.e. whole country, a state, a province or multiple neighbor cities, we considered the coordinates that resemble the point of symmetry of geometrical figure of that political region. By using an online calculator based on the international geomagnetic reference field data source [30], geomagnetic coordinates were calculated from geographical coordinates for each location in the year that MS prevalence was reported or in the nearest available time to that year. For example, geographical latitude of London is 51.5° N, whereas its geomagnetic latitude in 1985, the nearest time to the last MS prevalence report, was recorded as 53.66° N.

For any location, AMAG60 was determined by calculation of absolute value of numerical difference between its geomagnetic latitude from geomagnetic 60° latitude (GM60L). A same manner was done for calculation of AGRAPH60.

### Analyses

By using meta-regression analysis, the association of MS prevalence with geographical latitude, geomagnetic latitude, AMAG60 and AGRAPH60 were evaluated separately. In each analysis, we regarded MS prevalence of populations as dependent variables and geographical or geomagnetic related variables as independent variables. Logarithmic and linear models were tested for each variable and the best fitted model was selected for final inter-variable comparison. Models were compared by their *adjusted R*^*2*^ (coefficient of determination) and *standard error of estimate* (SEE).

In all meta-regression analyses, prevalence data were weighted with the square root of the number of prevalent cases. In all tests, a p-value lesser than 0.05 was regarded as significant. We did analyses by using IBM SPSS statistics (IBM®, New York, USA).

## Results

**Figure 3:**
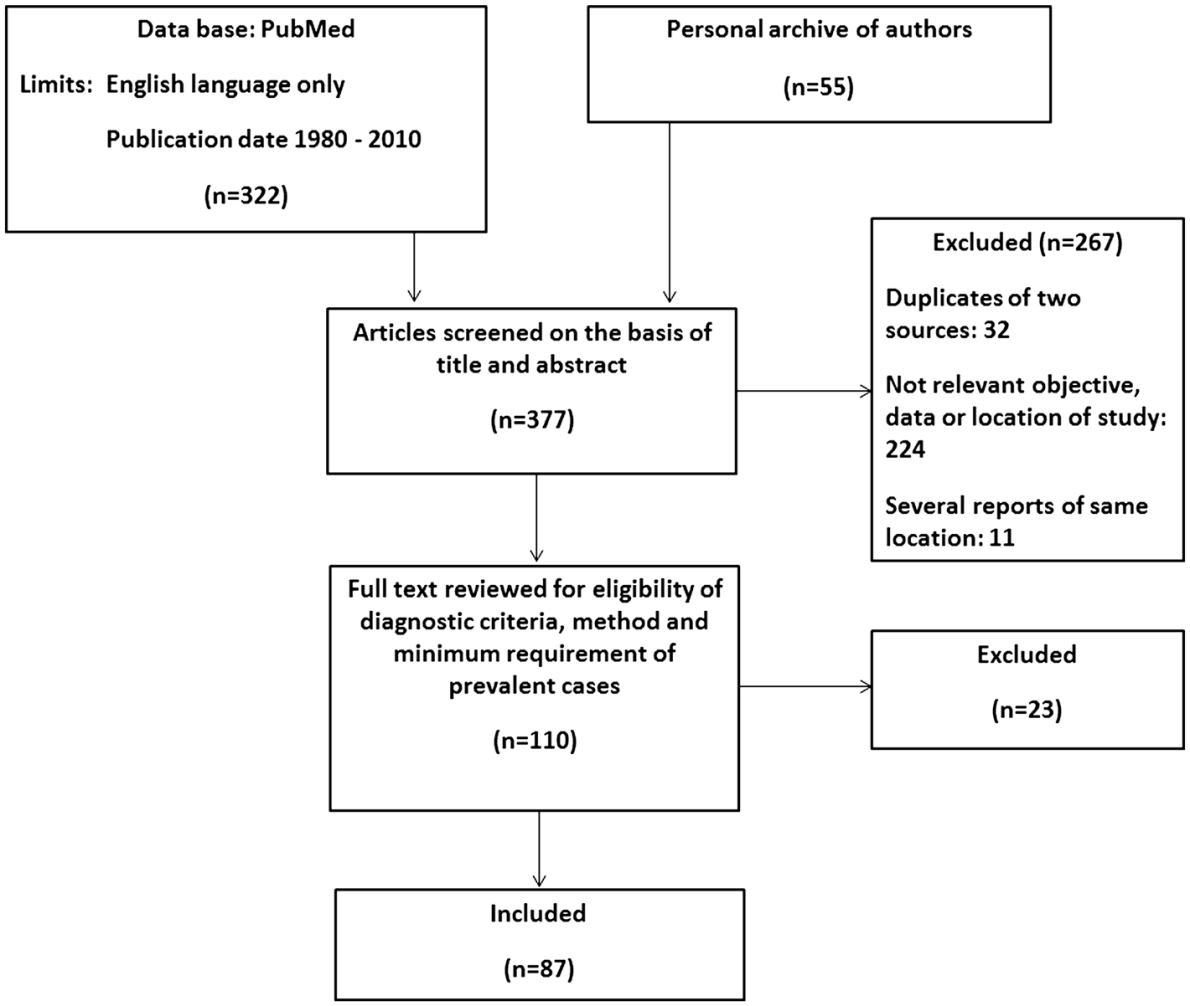
Flow diagram of study selection.

Among 377 retrieved and reviewed abstracts, full texts of 110 selected articles were read and according to our inclusion criteria, finally 87 papers from 24 western countries with 111 MS prevalence data were entered in the analyses. Of them, origin of 64 articles were from our search in the PubMed and 23 articles from authors' archive (Figure 3) (Table 1).

**Table 1:** Prevalence estimate data that were selected and entered for meta-regression analysis

**Location**	**Year**	**N. patients**	**Prevalence (per 10**^**5**^**)**	**GeoLat (Degree)**	**GeoMag (Degree)**	**AMAG60 (Degree)**	**AGRAPH60 (Degree)**	**Reference**
*North America*								
Canada								
Alberta	2004	11562	357	52	58.9	1.1	8	Warren et al. [31] †
Atlantic	2000	16032	350	49.3	59.3	0.7	10.7	Beck et al. [32] †
British Colombia	1982	4620	131	54	59.18	0.82	6	Sweeney et al. [33]
London (Ontario)	1983	190	94	42.59	53.21	6.79	17.41	Hader et al. [34]
Ontario	2000	33529	230	51	60.9	0.9	9	Beck et al. [32] †
Québec	2000	20551	180	52	62.27	2.27	8	Beck et al. [32] †
Saskatoon	2005	537	298	52.07	59.84	0.16	7.93	Hader and Yee [35]
Westlock	1991	23	200	54.09	61.11	1.11	5.91	Warren and Warren [36]
United States of America								
Colorado	1982	274	84	39.3	47.98	12.02	20.7	Nelson et al. [37]
Key West	1985	22	70	24.33	35	25	35.67	Helmick et al. [38]
Sugarcreek & Independence (Missouri)	2010	106	86	41.24	50.93	9.07	18.76	Noonan et al. [39]
Lorain county (Ohio)	2010	320	109	41.22	50.89	9.11	18.78	Noonan et al. [39]
Olmsted	2000	218	177	41.24	51.32	8.68	18.76	Mayr et al. [40]
Rochester	1984	102	173	44.02	53.24	6.76	15.98	Wynn et al. [41]
Texas	2000	182	42	31.3	40.26	19.74	28.7	Noonan et al. [39]
*Australasia*								
Australia								
Canberra	1996	155	49	35.16	42.74	17.26	24.84	Simmons et al. [42]
Newcastle	1996	79	59	32.55	39.86	20.14	27.45	Barnett et al. [43]
New South Wales	1981	1907	37	33	41.5	18.5	27	McLeod et al. [44]
South Australia	1981	378	28	30	39.77	20.23	30	McLeod et al. [44]
New Zealand	2010	2917	73.1					Taylor et al. [45] ‡
Auckland	2006	732	59	36.5	39.8	20.2	23.5	Taylor et al. [46]
Bay of plenty	2006	132	50	38.3	41.12	18.88	21.7	Taylor et al. [46]
Canterbury	2006	557	103	44.2	47.85	12.15	15.8	Taylor et al. [46]
Gisborne	2006	20	46.7	38.4	41.05	18.95	21.6	Taylor et al. [46]
Hawke Bay	2006	82	54.3	39	41.8	18.2	21	Taylor et al. [46]
Manawatu-Wanganui	2006	120	54	39.7	42.74	17.26	20.3	Taylor et al. [46]
Marlborough	2006	42	86.8	41.4	44.75	15.25	18.6	Taylor et al. [46]
Nelson-Tasman	2006	75	77.7	41.17	44.6	15.4	18.83	Taylor et al. [46]
Northland	2006	82	50.8	35.5	38.9	21.1	24.5	Taylor et al. [46]
Otago	2006	234	119.3	44.45	48.45	11.55	15.55	Taylor et al. [46]
Southland	2006	148	134.6	45.5	49.74	10.26	14.5	Taylor et al. [46]
Taranaki	2006	72	66.8	39.2	42.46	17.54	20.8	Taylor et al. [46]
Waikato	2006	177	46.4	37.7	40.8	19.2	22.3	Taylor et al. [46]
Wellington	2006	383	86.2	41.18	44.36	15.64	18.82	Taylor et al. [46]
*Western Europe*								
Austria	2000	3420	98	47.2	46.94	13.06	12.8	Baumhackl et al. [47]
Belgium								
Flandern	1991	220	88	51	52.54	7.46	9	van Ooteghem et al. [48]
Denmark	2005	9377	154	56	55.95	4.05	4	Bentzen et al. [49]
England								
Cambridgshire	1993	347	119	52.2	54.16	5.84	7.8	Robertson et al. [50]
Devon	2001	409	118	50.45	53.09	6.91	9.55	Fox et al. [51]
East Angelia	1990	374	112	52.3	54.49	5.51	7.7	Mumford et al. [52]
Guernsey	1993	53	95	49.28	51.81	8.19	10.72	Sharpe et al. [53]
Jersey	1993	95	120	49.2	51.69	8.31	10.8	Sharpe et al. [53]
Leeds	1996	712	84	53.5	55.67	4.33	6.5	Ford et al. [54]
London	1984	195	115	51.5	53.66	6.34	8.5	Williams and McKeran [55]
Northern East Angelia	1995	449	118	51.5	53.65	6.35	8.5	Robertson et al. [56]
Rochdale	1989	200	96	53.38	55.77	4.23	6.62	Shepherd and Summers [57]
Southampton	1987	384	92	50.55	52.97	7.03	9.45	Roberts et al. [58]
Suffolk	1988	58	153	52.18	54.05	5.95	7.82	Lockyer [59]
Sussex	1991	665	111	51.03	53.14	6.86	8.97	Rice-Oxley et al. [60]
Finland								
Central Finland	2000	277	105	62.5	59.31	0.69	2.5	Sarasoja et al. [61]
Sienajoki	1993	398	186	62.45	59.78	0.22	2.45	Sumelahti et al. [62]
Ussima	1993	1380	92	60.12	57.3	2.7	0.12	Sumelahti et al. [62]
Vaasa	1993	199	108	63.09	60.53	0.53	3.09	Sumelahti et al. [62]
France								
Lorraine	2004	2718	120	49	49.93	10.07	11	Debouverie et al. [63]
Germany								
Rostock	1983	193	89	54.15	53.81	6.19	5.85	Meyer-Rienecker and Buddenhagen [64]
South Lower Saxony	1986	222	83	52.63	52.84	7.16	7.37	Poser et al. [65]
Southern Hesse	1980	324	52	49.8	50.28	9.72	10.2	Lauer et al. [66]
Iceland	1989	252	100	65	62.9	2.9	5	Benedikz et al. [67]
Ireland								
Donegal	2001	240	185	54.5	57.77	2.23	5.5	McGuigan et al. [68]
Wexford	2001	126	121	52.2	55.28	4.72	7.8	McGuigan et al. [68]
Italy								
Alghero	1980	44	59	40.34	44.5	15.5	19.66	Rosati et al. [69]
Aosta	1989	36	39	45.44	46.41	13.59	14.56	Sironi et al. [70]
Bagheria	1994	25	49	38.05	38.07	21.93	21.95	Salemi et al. [71]
Barbagia	1981	32	78	40.56	41.3	18.7	19.44	Granieri et al. [72]
Caltanissetta	2002	101	166	37.48	37.37	22.63	22.52	Grimaldi et al. [73]
Catania	1995	195	58	37.5	37.21	22.79	22.5	Nicoletti et al. [74]
Enna	1995	34	120	37.34	37.22	22.78	22.66	Grimaldi et al. [75]
Ferrara	2003	423	121	44.53	44.9	15.1	15.47	Granieri et al. [76]
Genoa	1997	857	85	44.25	44.99	15.01	15.75	Solaro et al. [77]
L’Aquila city	1984	22	34	42	42.2	17.8	17.78	Salerni et al. [78]
L’Aquila Province	1996	158	56	42.22	42.13	17.87	17.78	Totaro et al. [79]
Modena	1990	404	39	44.4	44.73	15.27	15.6	Guidetti et al. [80]
Monreale	2000	21	71	38.05	38.08	21.92	21.95	Ragonese et al. [81]
North Western Sardinia	1991	276	103	40.12	40.93	19.07	19.88	Rosati et al. [82]
Padova	1999	667	81	45.4	45.44	14.56	14.6	Ranzato et al. [83]
Salerno	2005	186	72	40.67	40.31	19.69	19.33	Iuliano and Napoletano [84]
Nuoro	1993	394	144	40.19	40.96	19.04	19.81	Casetta et al. [85]
Sassari	1997	686	144	40.44	41.31	18.69	19.56	Pugliatti et al. [86]
Malta	1999	63	13	35.5	35.39	24.61	24.5	Dean et al. [87]
Northern Ireland	2004	370	200	54.4	57.28	2.72	5.6	Gray et al. [88]
Norway								
Hordaland	2003	666	151	60.15	60.53	0.53	0.15	Grytten et al. [89]
Møre og Romsdal	1985	159	75	62.3	62.42	2.42	2.3	Midgard et al. [90]
Nord Trondelag	2000	208	164	64.43	63.53	3.53	4.43	Dahl et al. [91]
Oslo	2005	759	170	59.56	59.3	0.7	0.44	Smestad et al. [92]
Troms and Finmark	1993	184	73	69.4	66.8	6.8	9.4	Gronlie et al. [93]
Vestfold	1983	163	86	59.25	58.8	1.2	0.75	Edland et al. [94]
Portugal								
Santarem	1998	29	46	39.14	43	17	20.86	De Sa et al. [95]
San Marino (Republic of)	2005	50	167	43.56	43.56	16.44	16.44	Granieri et al. [96]
Scotland								
Glasgow	2002	245	145	55.53	58.06	1.94	4.47	Murray et al. [97]
Lothian and Border	1995	1613	203	55.55	57.93	2.07	4.45	Rothwell and Charlton [98]
Orkney	1983	37	193	59	61.29	1.29	1	Cook et al. [99]
Shetland	1986	40	184	60.3	62.18	2.18	0.3	Cook et al. [100]
Tayside	1996	727	184	55.87	58.41	1.59	4.13	Forbes et al. [101]
Spain								
Alcoy	1988	23	17	38.42	40.92	19.08	21.58	Matias-Guiu et al. [102]
Bajo Aragon	2003	44	75	41	43.43	16.57	19	Modrego and Pina [103]
Canary Islands	1998	34	42	28	33.26	26.74	32	Hernandez [104]
Gijon	1994	22	65	43.32	46.62	13.38	16.68	Uria et al. [105]
Las Palmas	2002	64	61	28.6	33.83	26.17	31.4	Aladro et al. [106]
Menorca (Balearic Islands)	1996	46	69	40	41.68	18.32	20	Casquero et al. [107]
Mostoles	1998	85	43	40.19	43.17	16.83	19.81	Benito-Leon et al. [108]
Northern Calatayud,	1995	34	58	41.21	43.84	16.16	18.79	Pina et al. [109]
Osona	1991	42	58	41.7	43.78	16.22	18.3	Bufill et al. [110]
Santiago de Compostela	2003	71	79	42.53	46.18	13.82	17.47	Ares et al. [111]
Teruel	1996	46	32	40.21	42.82	17.18	19.79	Modrego Pardo et al. [112]
Valladolid	1997	54	58	41.39	44.57	15.43	18.61	Tola et al. [113]
Sweden	2008	17485	189	62	60.7	0.7	2	Ahlgren et al. [114]
Varmland	2002	580	170	59.48	58.69	1.31	0.52	Bostrom et al. [115]
Vasterbotten	1997	399	154	64.36	61.98	1.98	4.36	Sundstrom et al. [116]
Switzerland								
Berne	1986	1016	110	46.57	47.53	12.47	13.43	Beer and Kesselring [117]
Wales								
South Glamorgan	1985	381	101	51.3	54.08	5.92	8.7	Swingler and Compston [118]
South-East	2005	620	146	51.3	53.79	6.21	8.7	Hirst et al. [119]

N. Patients: Number of patients; GeoLat: Geographic latitude; GeoMag: Geomagnetic latitude; AMAG60: Angular distance to geomagnetic 60 latitude; AGRAPH60: Angular distance to geographic 60 latitude.

† Studies that did not use a determined MS diagnostic criteria.

‡ It was the latest report of MS prevalence in New Zealand (the entire country) that we found in the PubMed. But we found that their study result of prevalence in each region of that country was published previously by MS society of New Zealand [46]. We entered the latter study [46] results in our analyses.

**Figure 4:**
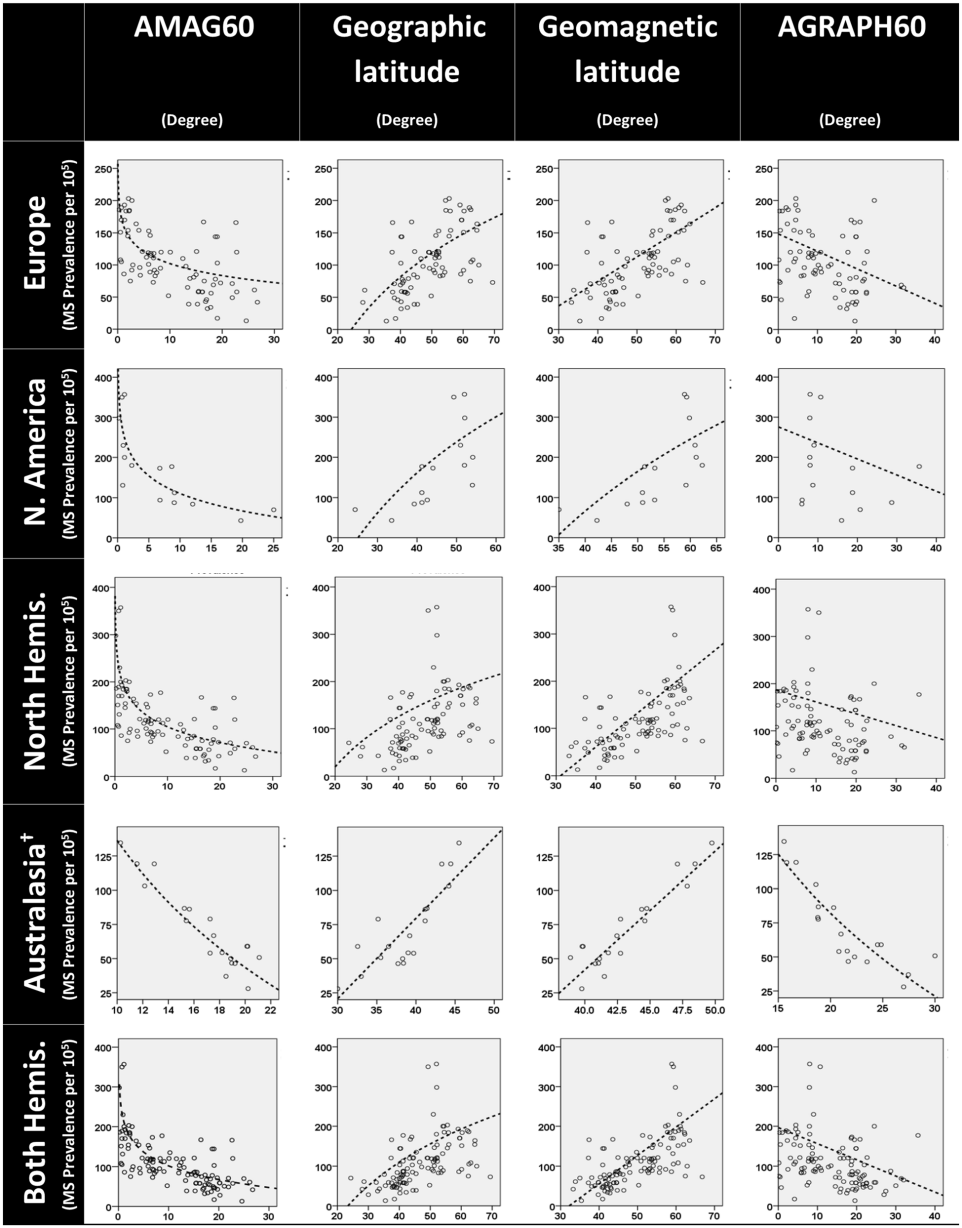
**MS Prevalence estimates by AMAG60, geographical latitude, geomagnetic latitude and AGARPH60.** AMAG60: Angular distance to geomagnetic 60° latitude; AGRAPH60: Angular distance to geographic 60° latitude; N. America: North America; North hemis: North hemisphere; Both hemis: Both hemispheres. † Australasia comprises Australia and New Zealand. Dotted lines indicate line of regression.

Of 111 entered MS prevalence data, 19 data were from Australasia, including Australia and New Zealand, 77 from Europe and 15 data were from North America. We plotted MS prevalence estimate data against geographic and geomagnetic latitudes, AGRAPH60 and AMAG60, separated by continents. Results were summarized in Table 2 and Figure 4 (Additional file 2: Appendix 2, Additional file 3: Appendix 3, Additional file 4: Appendix 4, Additional file 5: Appendix 5 and Additional file 6: Appendix 6 include high resolution formats of Figure 4 separated by rows ). All latitudinal variables of study showed significant statistical correlation with MS prevalence. In each continent, geomagnetic latitude showed approximately identical or mildly better relationship with MS prevalence in comparison to geographic latitude. Their models had nearly equal adjusted R^2^ (AR^2^) and SEE. Model based on AMAG60 had always the best association with MS prevalence with the greatest AR^2^ and the least SEE. AGRAPH60 model showed the weakest association with MS prevalence in each continent, with the least AR^2^ and the greatest SEE.

**Table 2:** Coefficients of regression models

	**Europe**			**North America**			**North Hemisphere**			**Australasia†**			**Both hemispheres**		
	**R**	**AR**^**2**^	**SEE**	**R**	**AR**^**2**^	**SEE**	**R**	**AR**^**2**^	**SEE**	**R**	**AR**^**2**^	**SEE**	**R**	**AR**^**2**^	**SEE**
AMAG60	0.69	0.47	33.8	0.65	0.42	69.85	0.71	0.50	60	0.92	0.84	11.92	0.75	0.56	57.07
GeoLat	0.58	0.34	37.93	0.40	0.16	84.36	0.27	0.07	82	0.89	0.79	13.67	0.41	0.17	78.48
GeoMag	0.60	0.36	37.17	0.40	0.16	84.36	0.57	0.33	70	0.91	0.83	12.41	0.63	0.40	66.50
AGRAPH60	0.42	0.18	42.27	0.21	0.05	89.85	0.19	0.04	83	0.92	0.84	11.96	0.35	0.12	80.55

R: Coefficient of correlation; AR^2^: Adjusted R^2^ (coefficient of determination); SEE: Standard error of estimates; AMAG60: Angular distance to geomagnetic 60 latitude; AGRAPH60: Angular distance to geographic 60 latitude; GeoLat: Geographic latitude; GeoMag: Geomagnetic latitude.

† Australasia is a region that geopolitically comprises Australia and New Zealand.

When we merged the data of Europe and North America to check the ability of models to explain MS prevalence variations in north hemisphere, AMAG60 was the best model that could describe 50% of variations in MS prevalence in a logarithmic manner, with a very strong correlation and the least SEE. The relationship of geographic latitude and AGRAPH60 was weak and they could describe only 7% and 4% of MS prevalence variation of north hemisphere, respectively.

Merging the data of both hemispheres, models based on AGRAPH60, geographic latitude and geomagnetic latitude could explain 12%, 17% and 40% of variations in MS prevalence, respectively. Whereas, AMAG60 illustrated very strong association with MS prevalence and could describe 56% of its variations with the least SEE.

## Discussion

All latitudinal variables of study showed significant statistical association with MS prevalence. It was expected to some extent, due to the latitudinal dependent nature of MS. Our analyses confirmed that in each continent, the association of MS prevalence with geomagnetic latitude is equal or mildly better than geographic latitude. It should be noted that attention to the association of MS with geomagnetic *coordinates* has precedent. About 50 years ago Barlow noticed that geomagnetic latitude could give a better explanation about MS prevalence and some of its special distribution features, such as the cause of lower prevalence of MS in Japan in comparison to other location with identical geographical latitude [6]. However he paid attention to an important fact, unfortunately he never realized the critical role of geomagnetic ~60° latitude and possible role of geomagnetic *disturbances*. He suggested that the relation of MS with geomagnetic latitude is originated from cosmic-ray related production of radioactive atoms in atmosphere and their radiation effects [6]. Nevertheless his opinion is very close to ours in the basic epidemiological aspect, but our hypothesis and its descriptions about MS pathogenesis and special features are completely different from Barlow’s final assumptions. As his hypothesis about mutations due to radioactive effects of cosmic rays could not explain MS pathophysiology and behavior, the whole fact was neglected.

Our results indicated that in each continent, AMAG60 can give the best explanation about the variation of MS prevalence. However, result of Australasia, including Australia and New Zealand, was different from other continents and all latitudinal variables illustrated very strong association with MS distribution. They identically could describe about 80% of variation of MS prevalence. We regarded this as another confirmation for the effect of AMAG60. As is obvious in Figure 1, Australasia is located below geomagnetic ~60 latitude. Therefore, it seems rationale that MS distribution shows a linear gradient toward this critical line. This very linear arrangement causes that geomagnetic and geographic latitude are able to explain variation of MS prevalence like AMAG60. Geographic 60° latitude is also located beyond Australasia. Due to this reason, AGRAPH60 could describe 84% of MS prevalence in this continent.

Merging the data of continents, especially in north hemisphere, we found that AMAG60 can give the best explanation about MS prevalence throughout the world in comparison to other latitudinal variables. We regard this advantage of AMAG60 as strong evidence that the mysterious environmental risk factor for MS should be correlated to this line more than other geographical factors. We mentioned in introduction that Earth’s surface is subject to experience magnetic field disturbances up to about 2000 nT under the auroral oval area because of substorms. GM60L is the line that usually represents the border of this oval in the most frequent disturbed situation, i.e. Kp = 3 situation. Therefore, considering the supportive results of our meta-regression analyses, we suppose that GMD can be the best candidate to be the mysterious environmental risk factor for MS.

Beyond of being statistically associated to MS prevalence distribution, we believe that GMD hypothesis has the ability to describe other important features of MS. As the core of our hypothesis, we illuminated how GMD hypothesis may provide essential context for explaining MS pathophysiology at molecular and cellular level. In the following paragraphs, the ability of GMD to explain other features of MS will be discussed.

### MS prevalence in hemispheres

Parabolic gradient of MS prevalence in north hemisphere and linear gradient in the south hemisphere can be explained easily by GMD. As is obvious in Figure 1, there are many inhabitant areas under and beyond GM60L in the Europe and North America, while there are not any inhabitant lands beyond this line in the south hemisphere. If an important environmental factor for MS is related to this line, it would be reasonable that the disease shows parabolic and linear distribution in north and south hemisphere, respectively.

### Worldwide MS incidence and prevalence trend

When MS was framed by Charcot in mid-19th century, it was considered as a rare disease and a subject for case report [7]. During 20^th^ century, MS incidence and admission grew very fast. This change was interpreted by some researchers as “an epidemic of recognition rather than the effect of altered biological factors” [7]. But from 1930s, an increase of MS incidence was reported initially from high latitudes in north hemisphere like Iceland that became notable from 1945 to 1954 [120]. Simultaneously, an identical trend of changes was reported from South Africa in south hemisphere [121]. Then, remarkable increase of MS incidence and prevalence were reported from various locations such as Denmark, Faroe Islands, Norway and Australia after 1960 [44,120].

Afterwards, however higher latitudes experienced a decreasing trend of incidence for a short period after 1965–70 [120,122], increasing incidence and prevalence was reported from Scotland, United Kingdom and Netherlands. Such a course of events could not be explained by survival changes or case ascertainment issues easily [120].

Very interestingly, mentioned course of MS prevalence and incidence can be explained by GMD. Fortunately, solar activity has been observed by means of regular recording of sunspot numbers since 1700 [123]. Sunspot numbers are correlated with solar cycles, solar magnetic activity and hence with the frequency and strength of GMD. Recently, Solanki et al. reconstructed sunspot numbers for the past 11,400 years. Their results illustrated an increasing and longstanding exceptional solar magnetic activity since 70 years ago that is unprecedented during past 8000 years [123]. Long-term analysis of recorded GMF activities revealed that GMDs have followed solar activity changes and have been increased, a phenomenon that is known as “centennial increase of geomagnetic activity” [124].

As a result, if MS is regarded a phenomenon related to GMD, it not only can explain why MS or reports of clinical manifestations resembling MS were rare in 19^th^ century and in medicine history of all previous centuries [7], but also it can clarify why MS incidence and prevalence have raised during 20^th^ century.

**Figure 5:**
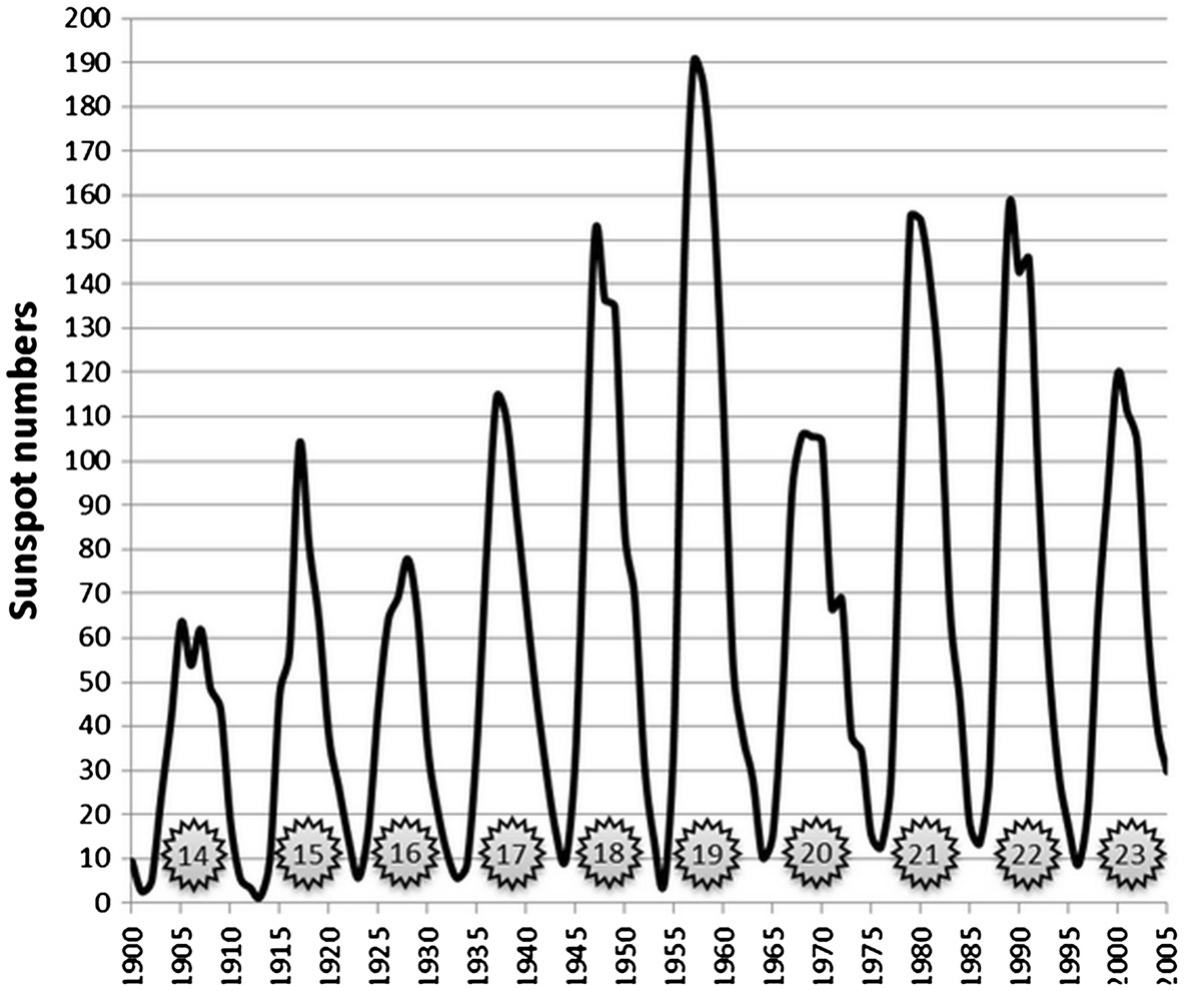
**Solar cycles and sunspot numbers.** Note: number in the stars indicates to the solar cycle number. Reproduced by the kind permission of National Geophysical Data Center [125].

Figure 5 shows mean yearly sunspot numbers. Since 1937 solar maximums started an increasing trend, reached to mean yearly sunspot number of 151 in 1947 and then registered a record in 1957. It was followed by a decrease in maximum sunspot number in the next cycle (20^th^ cycle), but backed to more than 150 spots in the maximums of the following cycles. Figure 2 shows that from 1933 to 1964, cycle average frequency of occurrence of Kp = 3 as well as other Kp situations had an increasing trend. Among 1944 to 1955, the average occurrences of Kp = 3 situation increased considerably and reached to its maximum by 1960. As mentioned, in Kp = 3 situation the edge of auroral oval is located over geomagnetic ~60° latitude, therefore, the disturbances in ground level magnetic field could be the cause of the increase in MS incidence and prevalence in the areas under and near to this line, relative to their angular distance to it. Regarding to these facts, GMD hypothesis has the potential ability to explain why MS incidence and prevalence in high latitudes increased from 1945–54 and dramatically by 1960. It also can be seen that average frequency of occurrences of Kp = 7 situation, that occurs in severe geomagnetic storms, increased about 2.3 folds in 1955–64 in comparison to 1933–44. Such severe storms not only expand the edge of auroral oval to about geomagnetic 50° latitude, equal to geographic 55° latitude in central Europe and to geographic 40° latitude in north America, but also cause global GMD that can explain why MS incidence and prevalence started to increase globally after 1960.

In both Figures 4 and 2, we can see that in 1965-76 (cycle 20), solar activity and frequency of all levels of GMD decreased temporarily. It can explain why MS incidence decreased in higher latitudes at this period.

### Gradual attenuation of latitudinal gradient of MS

In recent decades, increasing prevalence of MS in some low latitude areas like Italy, against the expected north–south gradient of MS [75,76], led to attenuation of previously prominent latitudinal gradient of MS [3]. An interesting finding about centennial increase of GMD can describe the reason. However centennial increase of GMD has had a latitudinal dependent nature and higher latitudes has experienced the largest disturbances because of proximity to auroral oval, long term studies have revealed surprisingly that the absolute amount of centennial increase of GMD in low-latitudes has been larger than mid-latitudes [124]. By this fact, it should not be surprising that the rate of MS prevalence rising in susceptible individuals of low-latitudes becomes even larger than individuals of mid-latitudes over the time, and consequently the prominent latitudinal gradient attenuates gradually.

### The effect of month of birth

According to the result of two large epidemiological studies, the risk of MS has associated to the month of birth. Birth in May for inhabitants of north hemisphere and November for south hemisphere have significantly related to higher risk of MS in adulthood [126,127]. Previously, it has been tried to explain this feature by means of maternal exposure to ultraviolet in the first trimester [127]. As CNS myelination occurs mainly in the third trimester, mostly from 29–39 gestational weeks [128,129], it seems more reasonable that the mysterious environmental factor should be related to this time. Consequently, for individuals born in May and November, the environmental factor that may be related to myelination time should occur at about mid-March and mid-September, respectively.

However it is very complicated and is far beyond the scope of this article, but it is well established that there are semiannual increases in GMD which take place near the time of equinoxes. In March, the earth reaches to the highest southern solar latitudes, in the region that is exposed to the fast solar winds [130]. At this time, however both hemispheres are affected, negative interplanetary magnetic field B_x_ component and positive dipole tilt of the earth cause favorable situation for northern hemisphere high latitude *reconnection phenomenon* that leads to accentuation of transpolar arcs and magnetic field disturbances predominantly in auroral oval of northern hemisphere [131]. In September, the situation is vice versa and reconnection phenomenon is facilitated in the southern hemisphere high latitudes [131]. If we assume that GMD can increase the risk of MS by affecting adaptive cell immunity and causing memory T-cells that will be activated in the future when identical temporary changes occur due to magnetic field disturbances, therefore, it could be hypothesized that genetically vulnerable individuals who were exposed to more GMD during their CNS myelination process, when various antigens of myelin structures are exposed and are prone to be recognized by immunity, will have increased risk for developing MS in the future. By this manner, GMD hypothesis not only can efficiently explain the relation of month of birth with the risk of MS, but also can describe why immigrants born in high-risk area mostly preserve the risk of their birthplace when immigrate to low-risk places. In the other hand, we hypothesize that genetically susceptible immigrants from low-risk to high-risk areas, however experience lower exposures in their fetal period that result in lower likelihood of production of memory T-cells against myelin structures, but will show increased MS risk [2] in their new residence area due to greater frequency of exposure to GMD that will increase the chance of being exposed to a matched GMD with their memory T-cells sensitivity.

Moreover, we predict that if studies about the effect of birth month on MS risk would be conducted in mid to low latitude geomagnetic areas, we would see the increasing risk attributed to both equinox time of spring and autumn, because these locations experience approximately identical increase in geomagnetic disturbances at these times.

### MS epidemics

Kurtzke et al. reported evidences of four epidemics of MS in small population of Faroe Islands during 1940–1991 [132]. They tried to explain these epidemics by defining a hypothetical pathogen, possibly introduced by British troops in 1945 [132]. Nevertheless, such a pathogen has not been found [2].

Considering the fact that solar magnetic activities and related GMDs during 20^th^ solar cycle were significantly lesser than previous and next cycles, and as a consequence assuming that they were not strong enough to cause MS in susceptible Faroese, we can hypothesize that what were seen in limited population of Faroe as separated MS epidemics, were reflections of solar magnetic activities and their related geomagnetic consequences on vulnerable individuals during 17^th^, 18^th^, 19^th^ and 21^st^ solar cycles. Albeit this statement needs to be tested by exact superposed epoch analysis of solar magnetic activities, GMDs and MS incidence in Faroe during mentioned period.

### Comparison with vitamin D hypothesis (VDH)

Finally and as a comparison, VDH has important weaknesses. We mentioned before that VDH cannot describe the cause of parabolic prevalence of MS. In fact, the relationship between the amount of solar ultraviolet B (UVB) penetration and latitude are complex, due to some factors such as differences in the thickness of atmosphere, cloud coverage and ozone cover situation. A recent modeling study has shown that the notion of latitudinal gradient of vitamin D levels in population is not accurate [133,134]. Accordingly, geophysical studies has confirmed that the amount of received UVB in a high latitude area like Canada, over 24 h during summer times, equals or even surpasses the received UVB at the equator [133].

In the other hand, VDH cannot explain chronobiological changes in MS incidence and prevalence. We know that vitamin D related rickets was endemic in many areas such as England in mid-17^th^ centuries. It was a common problem up to 1930 when finally by finding the cause and using cod liver oil and enough sun exposure, medicine overcame the disease [135]. Therefore, if MS actually is related to vitamin D deficiency, it is reasonable that we had records of more incidence and prevalence of MS or reported clinical manifestation that resembling MS before 1930. We know that it is in contrast with what has happened during history of MS. Ecological and genetic studies did not support the significant association between serum vitamin D level and MS, and observational studies did not find strong direct evidences of vitamin D effects on MS incidence [133]. Moreover, VDH cannot exactly illuminate the cause of recent attenuation of latitudinal gradient of MS prevalence and some phenomenon like MS epidemics [4].

### Magnetic resonance imaging (MRI): should we be concerned about it?

Nowadays MRI is the mainstay of diagnosis and follow- up of MS patients. During preparation of this manuscript and regarding used facts and mechanisms to construct and describe our hypothesis, we realized that the presence of the words “magnetic” and “resonance” in the name of MRI will inevitably evoke questions about its safety for MS patients. Authors, as designers of GMD hypothesis, cannot answer such questions definitely at present time. The main cause of uncertainty in this issue originates from the fact that we are not aware about the exact mechanism that GMD or other magnetic fields may probably elicit an immune response in CNS. Undoubtedly, the answer to this matter is completely dependent to the mechanism of such effect. For example, if future studies confirm that GMD may elicit immune response in human body mainly by changing lymphocyte Ca^+2^, as was described in introduction and subsection of “the hypothesis”, then with a high probability, at least in the case of inducing cyclotron resonance in Ca^2+^, we should not be concerned about MRI.

However MRI works by inducing cyclotron resonance, but the target of this technic is hydrogen nucleus. It is a physical fact that resonant frequency of atomic nucleus of any element is different from others and is related to its electric charge, atomic mass and albeit the strength of exerted magnetic field. In quantum mechanics, this resonant frequency can be calculated by means of Larmor equation. The Larmor equation is ω_0_ = yB_0_. Where ω_0_ is the resonant frequency, y is a unique constant for any element that is called gyromagnetic ratio and B_0_ is the strength of external magnetic field. Gyromagnetic ratio of hydrogen is 42.58 mega Hertz per Tesla (MHz/T), while this ratio is 2.86 MHz/T for calcium. Therefore, in magnetic fields of clinical MRI, i.e. 1.5 and 3 Tesla, resonant frequencies of hydrogen are about 63 and 127 MHz, respectively. While these frequencies for calcium is about 4 and 8 MHz, respectively. As clinical MRI appliances use pulse frequencies of 60 MHz up to 128 MHz [136], their pulse frequencies are not match with calcium nucleus to elicit cyclotron resonance in them. It is the physical basis of a known fact in clinical practice that MRI is not the appropriate technic for imaging calcified tissues or bony structures.

In contrast, if future studies confirm GMD hypothesis and show that the mechanism of GMD effects is related to its impact on brain magnetosomes and their magnetite contents, that physically are sensitive to magnetic fields, then the safety of MRI for MS patients should necessarily be revaluated regarding to this matter.

In conclusion, the recommendation of authors at present time is to use MRI only for evaluating the presence of demyelinating lesions at the first attack or eventually for confirmation of dissemination of lesions in time for necessary cases, and then to follow-up MS patient clinically and to avoid ordering repeated MRI for this aim as far as possible.

### Limitations

However ecological study is a very suitable and inexpensive way for hypothesis evaluation in large scale and in population level, but it should be reminded that such studies are subject to be impressed by ecological fallacy. In the time of interpretation of the result, it should be noted that findings related to aggregate population may not always be applicable to individuals.

Other limitation of the study is its limitation in regarding the exact amount and time of exposure of population to GMD. Geomagnetic coordinates and experienced GMD of any locations change over time. However we determined AMAG60 of any location in the nearest time of its MS prevalence study and considered it as a variable related to the amount of experienced GMD by population, but we did not know the actual time in the life that experiencing GMD may affect individual susceptible for MS. It may be during fetal time, neonatal period, childhood or adulthood.

Nevertheless we provided evidences of how GMD possibly can provide essential components for causing MS, but it should be noticed that all of these evidences are from *in vitro* studies. In the other hand, however we assumed that there may be a genetical basis that can be the cause of sensitivity of susceptible individuals to GMD, such a genetic basis, except than a new finding about human cryptochrome CRY2 gene [137], was not confirmed or evaluated up to now.

Another important limitation of GMD hypothesis, at least at present time, is related to the issues of difference among sexes in the case of MS incidence and prevalence, and other related factors such as the effect of pregnancy or post-partum period on alteration of MS attacks. The main cause of inability of GMD hypothesis to provide a reasonable description about these issues originates from the lack of information about the exact mechanism of the effect of GMD. There are evidences that hormones, for example melatonin, may have a role in the response of brain to GMD [137]. But, we could not find such studies about sexual hormones. Therefore, providing probable explanations about these phenomena is dependent to future studies in this field.

## Conclusions

We described how GMD can explain main issues about MS. GMD hypothesis not only has the ability to provide possible explanation about MS in cellular level, but also has the ability to clarify the cause of relapsing-remitting nature, chronobiology, latitudinal prevalence, alterations in MS distribution and some phenomenon like the birth month effect and MS epidemics. It also can solve the puzzle of longstanding failure of finding the mysterious environmental cause of MS by biochemical marker dependent technics and give us the chance to make some general prediction about the disease activity, because space-weather situations and GMDs are relatively predictable.

It was just a preliminary evaluation of this hypothesis with limitation related to ecological study. At this time the main shortcoming of GMD hypothesis is the lack of direct evidences. Regarding to the provided answers by GMD for important issues of MS, we believe that our hypothesis deserves to be considered for further individual based validation studies. Like other scientific hypothesis, there is the possibility that future studies do not confirm GMD hypothesis. Nonetheless, the importance of GM60L should not be neglected and other possible environmental factors that may be related to this line and MS should be evaluated.

## Abbreviations

AR^2^: Adjusted R square; AGRAPH60: Angular distance to geographical 60 latitude; AMAG60: Angular distance to geomagnetic 60 latitude; Ap: Planetary A index; CNS: Central nervous system; GM60L: Geomagnetic 60 latitude; GMD: Geomagnetic disturbances; GMF: Geomagnetic field; Kp: Planetary K index; MHC: Major histocompatibility complex; MHz/T: Mega Hertz per Tesla; MS: Multiple sclerosis; SEE: Standard error of estimate; VLMF: Very low magnetic field; VDH: Vitamin D hypothesis.

## Competing interests

Both Authors declare that they have no competing interest.

## Authors’ contributions

SAS was the main designer of GMD hypothesis. FA was the researcher who retrieved MS prevalence studies according to study inclusion criteria. Both authors contributed to design and execution of this survey. Both authors participated in interpretation of the results and writing the article. Both authors approved the final report.

## Pre-publication history

The pre-publication history for this paper can be accessed here:

http://www.biomedcentral.com/1471-2377/12/100/prepub

## Supplementary Material

Additional file 1 **Appendix 1. **Search strategy. Click here for file

Additional file 2 **Appendix 2.** High resolution format of MS prevalence data of Europe from Figure 4**.** Click here for file

Additional file 3 **Appendix 3.** High resolution format of MS prevalence data of North America from Figure 4. Click here for file

Additional file 4 **Appendix 4.** High resolution format of MS prevalence data of North hemisphere from Figure 4. Click here for file

Additional file 5 **Appendix 5.** High resolution format of MS prevalence data of Australasia from Figure 4. Click here for file

Additional file 6 **Appendix 6.** High resolution format of MS prevalence data of both hemispheres from Figure 4. Click here for file
