# Supplementary material for: Geomagnetic disturbances may be environmental risk factor for multiple sclerosis: an ecological study of 111 locations in 24 countries
Source: BMC Neurol. 2012 Sep 24;12:100. doi: 10.1186/1471-2377-12-100 (PMC3488506; doi:10.1186/1471-2377-12-100)
Supplement: Additional file 1 — Appendix 1. Search strategy. [file 1471-2377-12-100-S1.docx]

Appendix 1. Search strategy

Data source: PubMed

1. “Multiple Sclerosis”[Title]
2. “Prevalence”[Title]
3. “Epidemiology”[Title]
4. ((#1) AND #2) OR ((#1) AND #3)
5. (#4) AND ("1980"[Date - Publication] : "2010/12/31"[Date - Publication])
6. English [Language]
7. ((#5) AND #6)
