# Supplementary material for: Geomagnetic disturbances may be environmental risk factor for multiple sclerosis: an ecological study of 111 locations in 24 countries
Source: BMC Neurol. 2012 Sep 24;12:100. doi: 10.1186/1471-2377-12-100 (PMC3488506; doi:10.1186/1471-2377-12-100)
Supplement: Additional file 2 — Appendix 2. High resolution format of MS prevalence data of Europe from Figure 4. [file 1471-2377-12-100-S2.pdf]

Table 1. Prevalence estimate data that were selected and entered for meta-regression analysis.

Location	Year	N. patients	Prevalence (per 10^5)	GeoLat (Degree)	GeoMag (Degree)	AMAG60 (Degree)	AGRAPH60 (Degree)	Reference
**North America**								
**Canada**								
**Alberta**	2004	11562	357	52	58.9	1.1	8	Warren et al. [52] †
**Atlantic**	2000	16032	350	49.3	59.3	0.7	10.7	Beck et al. [53] †
**British Colombia**	1982	4620	131	54	59.18	0.82	6	Sweeney et al. [54]
**London (Ontario)**	1983	190	94	42.59	53.21	6.79	17.41	Hader et al. [55]
**Ontario**	2000	33529	230	51	60.9	0.9	9	Beck et al. [53] †
**Québec**	2000	20551	180	52	62.27	2.27	8	Beck et al. [53] †
**Saskatoon**	2005	537	298	52.07	59.84	0.16	7.93	Hader and Yee [56]
**Westlock**	1991	23	200	54.09	61.11	1.11	5.91	Warren and Warren [57]
**United States of America**								
**Colorado**	1982	274	84	39.3	47.98	12.02	20.7	Nelson et al. [58]
**Key West**	1985	22	70	24.33	35	25	35.67	Helmick et al. [59]
**Sugarcreek & ind] Missouri**	2010	106	86	41.24	50.93	9.07	18.76	Noonan et al. [60]
**Lorain county (Ohio)**	2010	320	109	41.22	50.89	9.11	18.78	Noonan et al. [60]
**Olmsted**	2000	218	177	41.24	51.32	8.68	18.76	Mayr et al. [61]
**Rochester**	1984	102	173	44.02	53.24	6.76	15.98	Wynn et al. [62]
**Texas**	2000	182	42	31.3	40.26	19.74	28.7	Noonan et al. [60]
**Australasia**								
**Australia**								
**Canberra**	1996	155	49	35.16	42.74	17.26	24.84	Simmons et al. [63]
**Newcastle**	1996	79	59	32.55	39.86	20.14	27.45	Barnett et al. [64]
**New South Wales**	1981	1907	37	33	41.5	18.5	27	McLeod et al. [32]
**South Australia**	1981	378	28	30	39.77	20.23	30	McLeod et al. [32]
**New Zealand**	2010	2917	73.1					Taylor et al. [65] ‡
**Auckland**	2006	732	59	36.5	39.8	20.2	23.5	Taylor et al. [66]
**Bay of plenty**	2006	132	50	38.3	41.12	18.88	21.7	Taylor et al. [66]
**Canterbury**	2006	557	103	44.2	47.85	12.15	15.8	Taylor et al. [66]

**Gisborne**	2006	20	46.7	38.4	41.05	18.95	21.6	Taylor et al. [66]
**Hawke Bay**	2006	82	54.3	39	41.8	18.2	21	Taylor et al. [66]
**Manawatu-Wanganui**	2006	120	54	39.7	42.74	17.26	20.3	Taylor et al. [66]
**Marlborough**	2006	42	86.8	41.4	44.75	15.25	18.6	Taylor et al. [66]
**Nelson-Tasman**	2006	75	77.7	41.17	44.6	15.4	18.83	Taylor et al. [66]
**Northland**	2006	82	50.8	35.5	38.9	21.1	24.5	Taylor et al. [66]
**Otago**	2006	234	119.3	44.45	48.45	11.55	15.55	Taylor et al. [66]
**Southland**	2006	148	134.6	45.5	49.74	10.26	14.5	Taylor et al. [66]
**Taranaki**	2006	72	66.8	39.2	42.46	17.54	20.8	Taylor et al. [66]
**Waikato**	2006	177	46.4	37.7	40.8	19.2	22.3	Taylor et al. [66]
**Wellington**	2006	383	86.2	41.18	44.36	15.64	18.82	Taylor et al. [66]
**Western Europe**								
**Austria**	2000	3420	98	47.2	46.94	13.06	12.8	Baumhackl et al. [67]
**Belgium**								
**Flandern**	1991	220	88	51	52.54	7.46	9	van Ooteghem et al. [68]
**Denmark**	2005	9377	154	56	55.95	4.05	4	Bentzen et al. [69]
**England**								
**Cambridgshire**	1993	347	119	52.2	54.16	5.84	7.8	Robertson et al. [70]
**Devon**	2001	409	118	50.45	53.09	6.91	9.55	Fox et al. [71]
**East Angelia**	1990	374	112	52.3	54.49	5.51	7.7	Mumford et al. [72]
**Guernsey**	1993	53	95	49.28	51.81	8.19	10.72	Sharpe et al. [73]
**Jersey**	1993	95	120	49.2	51.69	8.31	10.8	Sharpe et al. [73]
**Leeds**	1996	712	84	53.5	55.67	4.33	6.5	Ford et al. [74]
**London**	1984	195	115	51.5	53.66	6.34	8.5	Williams and McKeran [75]
**Northern East Angelia**	1995	449	118	51.5	53.65	6.35	8.5	Robertson et al. [76]
**Rochdale**	1989	200	96	53.38	55.77	4.23	6.62	Shepherd and Summers [77]
**Southampton**	1987	384	92	50.55	52.97	7.03	9.45	Roberts et al. [78]
**Suffolk**	1988	58	153	52.18	54.05	5.95	7.82	Lockyer [79]
**Sussex**	1991	665	111	51.03	53.14	6.86	8.97	Rice-Oxley et al. [80]
**Finland**								
**Central Finland**	2000	277	105	62.5	59.31	0.69	2.5	Sarasoja et al. [81]
**Sienajoki**	1993	398	186	62.45	59.78	0.22	2.45	Sumelahti et al. [82]
**Ussima**	1993	1380	92	60.12	57.3	2.7	0.12	Sumelahti et al. [82]
**Vaasa**	1993	199	108	63.09	60.53	0.53	3.09	Sumelahti et al. [82]

**France**								
**Lorraine**	2004	2718	120	49	49.93	10.07	11	Debouverie et al. [83]
**Germany**								
**Rostock**	1983	193	89	54.15	53.81	6.19	5.85	Meyer-Rienecker and Buddenhagen [84]
**South Lower Saxony**	1986	222	83	52.63	52.84	7.16	7.37	Poser et al. [85]
**Southern Hesse**	1980	324	52	49.8	50.28	9.72	10.2	Lauer et al. [86]
**Iceland**	1989	252	100	65	62.9	2.9	5	Benedikz et al. [87]
**Ireland**								
**Donegal**	2001	240	185	54.5	57.77	2.23	5.5	McGuigan et al. [88]
**Wexford**	2001	126	121	52.2	55.28	4.72	7.8	McGuigan et al. [88]
**Italy**								
**Alghero**	1980	44	59	40.34	44.5	15.5	19.66	Rosati et al. [89]
**Aosta**	1989	36	39	45.44	46.41	13.59	14.56	Sironi et al. [90]
**Bagheria**	1994	25	49	38.05	38.07	21.93	21.95	Salemi et al. [91]
**Barbagia**	1981	32	78	40.56	41.3	18.7	19.44	Granieri et al. [92]
**Caltanissetta**	2002	101	166	37.48	37.37	22.63	22.52	Grimaldi et al. [93]
**Catania**	1995	195	58	37.5	37.21	22.79	22.5	Nicoletti et al. [94]
**Enna**	1995	34	120	37.34	37.22	22.78	22.66	Grimaldi et al. [36]
**Ferrara**	2003	423	121	44.53	44.9	15.1	15.47	Granieri et al. [37]
**Genoa**	1997	857	85	44.25	44.99	15.01	15.75	Solaro et al. [95]
**L'Aquila city**	1984	22	34	42	42.2	17.8	17.78	Salerni et al. [96]
**L'Aquila Province**	1996	158	56	42.22	42.13	17.87	17.78	Totaro et al. [97]
**Modena**	1990	404	39	44.4	44.73	15.27	15.6	Guidetti et al. [98]
**Monreale**	2000	21	71	38.05	38.08	21.92	21.95	Ragonese et al. [99]
**North Western Sardinia**	1991	276	103	40.12	40.93	19.07	19.88	Rosati et al. [100]
**Padova**	1999	667	81	45.4	45.44	14.56	14.6	Ranzato et al. [101]
**Salerno**	2005	186	72	40.67	40.31	19.69	19.33	Iuliano and Napoletano [102]
**Nuoro**	1993	394	144	40.19	40.96	19.04	19.81	Casetta et al. [103]
**Sassari**	1997	686	144	40.44	41.31	18.69	19.56	Pugliatti et al. [104]
**Malta**	1999	63	13	35.5	35.39	24.61	24.5	Dean et al. [105]
**Northern Ireland**	2004	370	200	54.4	57.28	2.72	5.6	Gray et al. [106]
**Norway**								
**Hordaland**	2003	666	151	60.15	60.53	0.53	0.15	Grytten et al. [107]
**Møre og Romsdal**	1985	159	75	62.3	62.42	2.42	2.3	Midgard et al. [108]

**Nord Trondelag**	2000	208	164	64.43	63.53	3.53	4.43	Dahl et al. [109]
**Oslo**	2005	759	170	59.56	59.3	0.7	0.44	Smestad et al. [110]
**Troms and Finmark**	1993	184	73	69.4	66.8	6.8	9.4	Gronlie et al. [111]
**Vestfold**	1983	163	86	59.25	58.8	1.2	0.75	Edland et al. [112]
**Portugal**								
**Santarem**	1998	29	46	39.14	43	17	20.86	De Sa et al. [113]
**San Marino (Republic of)**	2005	50	167	43.56	43.56	16.44	16.44	Granieri et al. [114]
**Scotland**								
**Glasgow**	2002	245	145	55.53	58.06	1.94	4.47	Murray et al. [115]
**Lothian and Border**	1995	1613	203	55.55	57.93	2.07	4.45	Rothwell and Charlton [116]
**Orkney**	1983	37	193	59	61.29	1.29	1	Cook et al. [117]
**Shetland**	1986	40	184	60.3	62.18	2.18	0.3	Cook et al. [118]
**Tayside**	1996	727	184	55.87	58.41	1.59	4.13	Forbes et al. [119]
**Spain**								
**Alcoy**	1988	23	17	38.42	40.92	19.08	21.58	Matias-Guiu et al. [120]
**Bajo Aragon**	2003	44	75	41	43.43	16.57	19	Modrego and Pina [121]
**Canary Islands**	1998	34	42	28	33.26	26.74	32	Hernandez [122]
**Gijon**	1994	22	65	43.32	46.62	13.38	16.68	Uria et al. [123]
**Las Palmas**	2002	64	61	28.6	33.83	26.17	31.4	Aladro et al. [124]
**Menorca (Balearic Islands)**	1996	46	69	40	41.68	18.32	20	Casquero et al. [125]
**Mostoles**	1998	85	43	40.19	43.17	16.83	19.81	Benito-Leon et al. [126]
**Northern Calatayud,**	1995	34	58	41.21	43.84	16.16	18.79	Pina et al. [127]
**Osona**	1991	42	58	41.7	43.78	16.22	18.3	Bufill et al. [128]
**Santiago de Compostela**	2003	71	79	42.53	46.18	13.82	17.47	Ares et al. [129]
**Teruel**	1996	46	32	40.21	42.82	17.18	19.79	Modrego Pardo et al. [130]
**Valladolid**	1997	54	58	41.39	44.57	15.43	18.61	Tola et al. [131]
**Sweden**	2008	17485	189	62	60.7	0.7	2	Ahlgren et al. [132]
**Varmland**	2002	580	170	59.48	58.69	1.31	0.52	Bostrom et al. [133]
**Vasterbotten**	1997	399	154	64.36	61.98	1.98	4.36	Sundstrom et al. [134]
**Switzerland**								
**Berne**	1986	1016	110	46.57	47.53	12.47	13.43	Beer and Kesselring [135]

**Wales**								
**South Glamorgan**	1985	381	101	51.3	54.08	5.92	8.7	Swingler and Compston [136]
**South-East**	2005	620	146	51.3	53.79	6.21	8.7	Hirst et al. [137]

N. Patients: Number of patients; GeoLat: Geographic latitude; GeoMag: Geomagnetic latitude; AMAG60: Angular distance to geomagnetic 60º latitude; AGRAPH60: Angular distance to geographic 60º latitude.

† Studies that did not use a determined MS diagnostic criteria.

‡ It was the latest report of MS prevalence in New Zealand (the entire country) that we found in the PubMed. But we found that their study result of prevalence in each region of that country was published previously by MS society of New Zealand [66]. We entered the latter study [66] results in our analyses.
