# Supplementary material for: Geomagnetic disturbances may be environmental risk factor for multiple sclerosis: an ecological study of 111 locations in 24 countries
Source: BMC Neurol. 2012 Sep 24;12:100. doi: 10.1186/1471-2377-12-100 (PMC3488506; doi:10.1186/1471-2377-12-100)
Supplement: Additional file 6 — Appendix 6. High resolution format of MS prevalence data of both hemispheres from Figure 4. [file 1471-2377-12-100-S6.pdf]

	AMAG60 (Degree)	Geographic latitude (Degree)	Geomagnetic latitude (Degree)	AGRAPH60 (Degree)
**Australasia†** (MS Prevalence per 10^5)	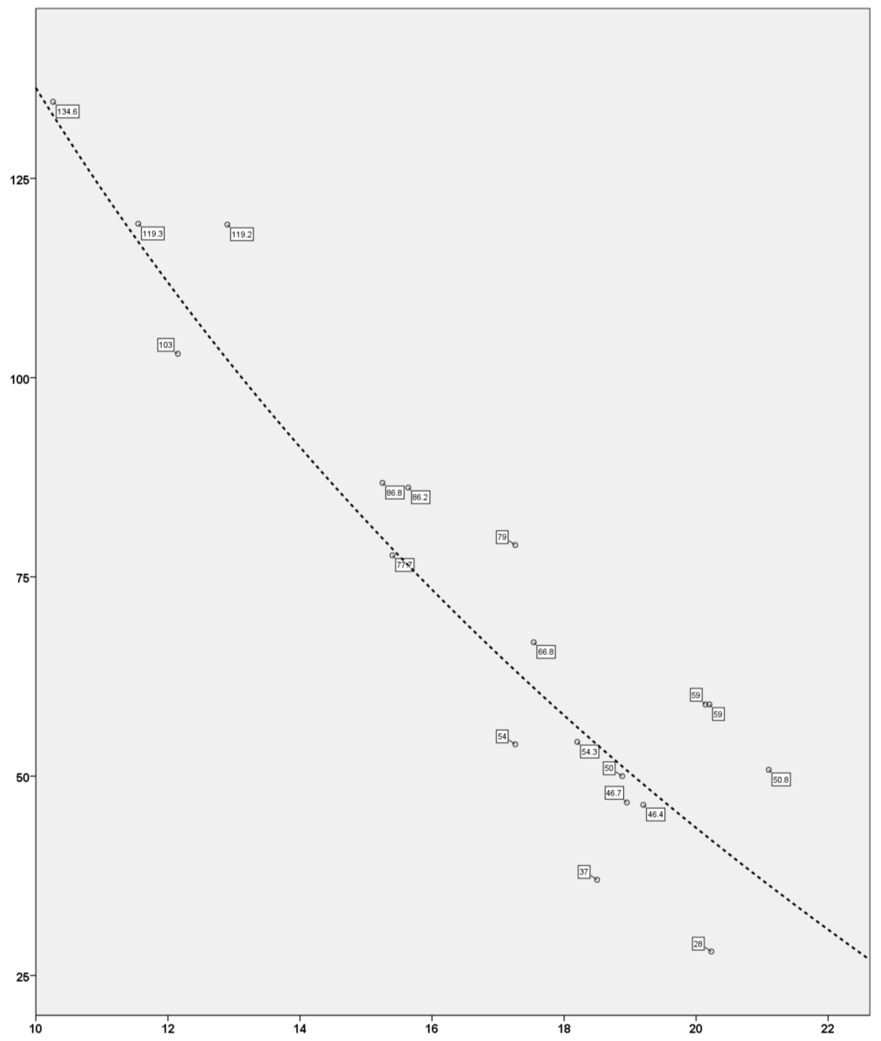	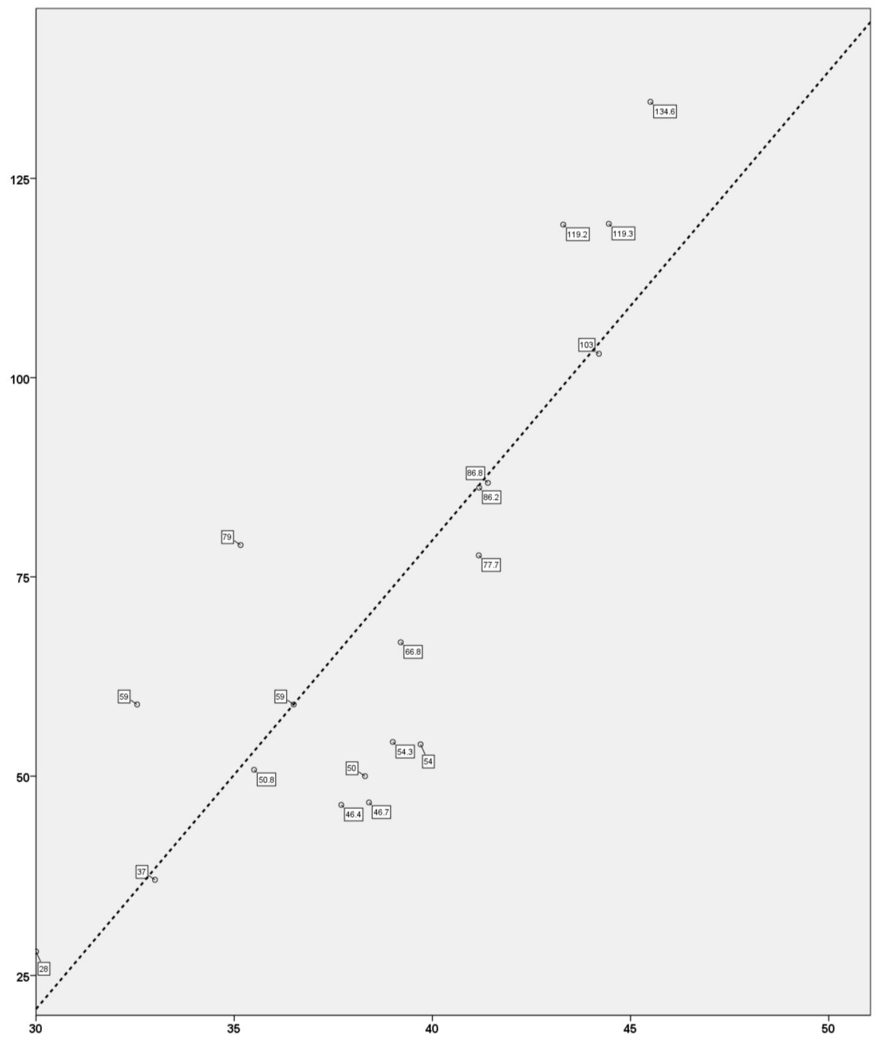	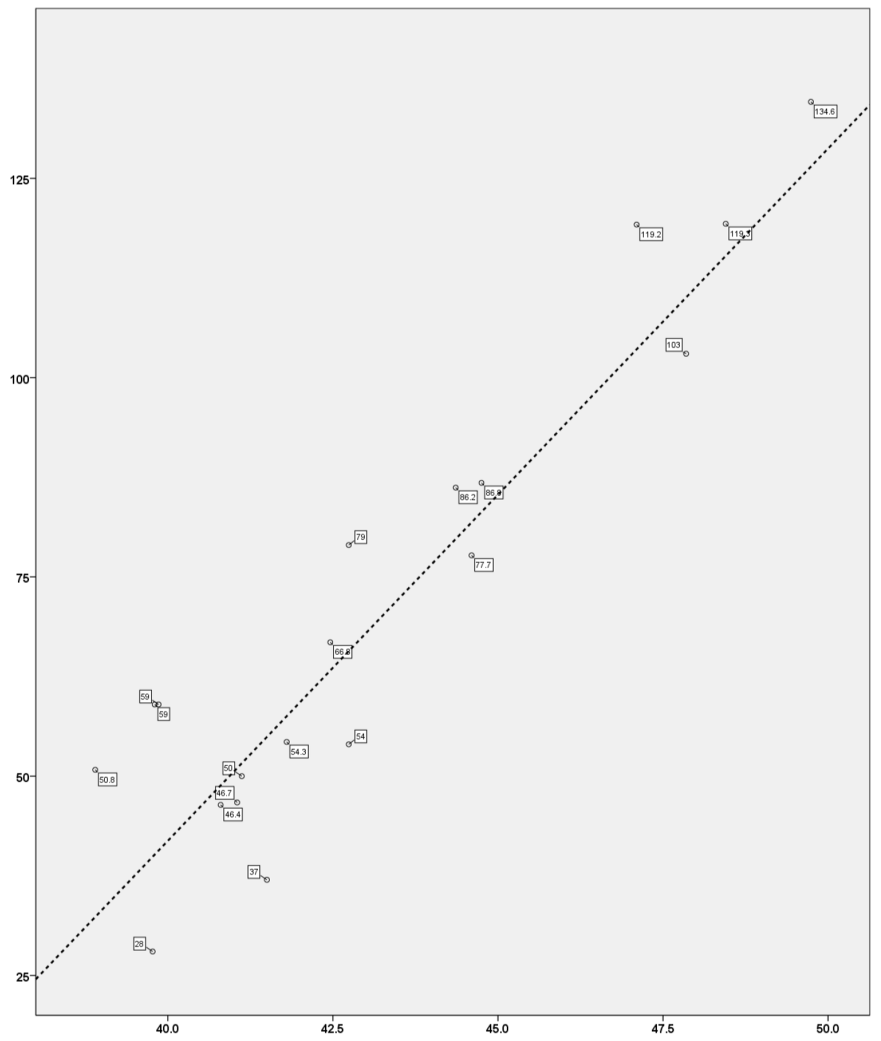	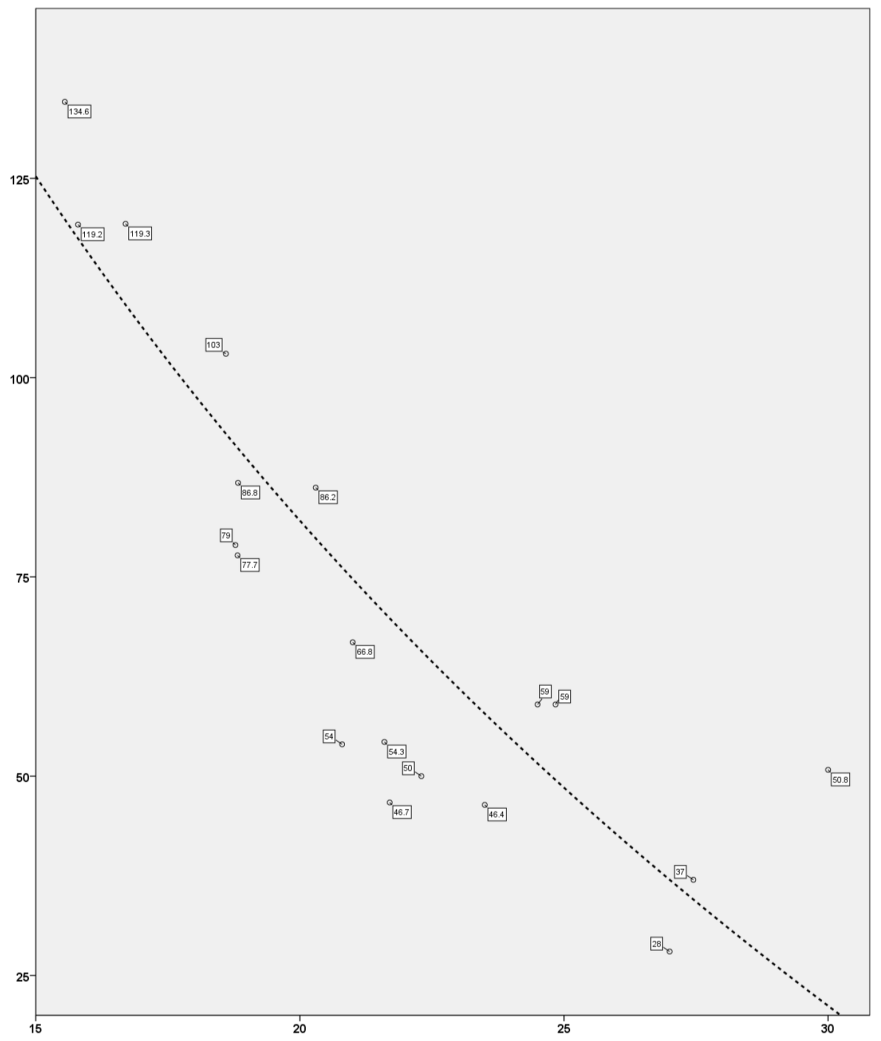

AMAG60: Angular distance to geomagnetic 60º latitude; AGRAPH60: Angular distance to geographic 60º latitude;

† Australasia comprises Australia and New Zealand. Dotted lines indicate line of regression.
